# A Microfluidic Platform for Correlative Live-Cell and Super-Resolution Microscopy

**DOI:** 10.1371/journal.pone.0115512

**Published:** 2014-12-29

**Authors:** Johnny Tam, Guillaume Alan Cordier, Štefan Bálint, Ángel Sandoval Álvarez, Joseph Steven Borbely, Melike Lakadamyali

**Affiliations:** ICFO-Institut de Ciències Fotòniques (ICFO), 08860, Castelledefels (Barcelona), Spain; University of Navarra, Spain

## Abstract

Recently, super-resolution microscopy methods such as stochastic optical reconstruction microscopy (STORM) have enabled visualization of subcellular structures below the optical resolution limit. Due to the poor temporal resolution, however, these methods have mostly been used to image fixed cells or dynamic processes that evolve on slow time-scales. In particular, fast dynamic processes and their relationship to the underlying ultrastructure or nanoscale protein organization cannot be discerned. To overcome this limitation, we have recently developed a correlative and sequential imaging method that combines live-cell and super-resolution microscopy. This approach adds dynamic background to ultrastructural images providing a new dimension to the interpretation of super-resolution data. However, currently, it suffers from the need to carry out tedious steps of sample preparation manually. To alleviate this problem, we implemented a simple and versatile microfluidic platform that streamlines the sample preparation steps in between live-cell and super-resolution imaging. The platform is based on a microfluidic chip with parallel, miniaturized imaging chambers and an automated fluid-injection device, which delivers a precise amount of a specified reagent to the selected imaging chamber at a specific time within the experiment. We demonstrate that this system can be used for live-cell imaging, automated fixation, and immunostaining of adherent mammalian cells *in situ* followed by STORM imaging. We further demonstrate an application by correlating mitochondrial dynamics, morphology, and nanoscale mitochondrial protein distribution in live and super-resolution images.

## Introduction

The crowded, intracellular environment is highly dynamic. Visualizing a specific subcellular process requires high spatial and high temporal resolution in combination with a molecular marker that specifically highlights the structure of interest. Recently, super-resolution microscopy methods have been developed which can image sub-cellular structures with nanoscale spatial resolution, breaking the classical diffraction limit in optical microscopy. One such technique is stochastic optical reconstruction microscopy (STORM) [1]. STORM belongs to a class of super-resolution methods that rely on single molecule localization [2], [3]. In single molecule localization microscopy, a photoswitchable fluorophore is used to label the structure of interest. These fluorophores are activated in sparse numbers such that their images are spatially separated, which allows each fluorophore to be precisely localized. The accumulation of many cycles of activation, localization, and deactivation results in a reconstructed image which reveals structures at a resolution well below the diffraction limit. STORM has enabled imaging of cellular morphology [4], protein organization [5], and sub-cellular structures such as mitochondria in fixed cells at spatial resolutions of up to 20 nm [6]. In addition, organelle and vesicle dynamics have been imaged in living cells at a spatial resolution of 30 nm and a temporal resolution of several seconds [7]–[9]. However, most cellular dynamics occur at much faster timescales (millisecond) and achieving both nanoscale spatial and millisecond temporal resolution is still very challenging using super-resolution microscopy methods [10]. To circumvent this problem, recently, we developed an all-optical correlative imaging approach that combines time-lapse live-cell microscopy with STORM to achieve both high temporal resolution and high spatial resolution, respectively [11]. This approach has enabled us to study cargo transport dynamics at the level of single microtubules, revealing how microtubule intersections impact motor-protein mediated transport. In principle, this approach can be extended to study other subcellular processes in which it is necessary to interpret dynamic information in the context of ultrastructural information. However, the technique requires precise delivery and removal of fluid from a sample that remains on the microscope stage for the duration of the experiment, a procedure that, when performed manually, is imprecise, labor-intensive, and time consuming.

To streamline and automate the sample preparation between live-cell imaging and super-resolution microscopy, we took advantage of PDMS-based microfluidic devices. While sophisticated options exist for automated immunostaining of mammalian cells [12], [13], we decided to use an approach with external valves and a very simple modular design that is cost-effective and easy to adopt. Our microfluidic chip for adherent mammalian cell culture yields miniaturized imaging chambers that are still large enough to contain a high number of cells that can form a confluent monolayer under healthy growth conditions. It is also compatible with live-cell time-lapse imaging, STORM, and other inverted microscopy techniques. We have carried out extensive optimization of sample preparation protocols that are specific to super-resolution imaging (fixation, immunostaining, and imaging buffers) to achieve optimal labeling densities, optimal photoswitching of fluorophores, and ultimately, optimal resolution in the reconstructed STORM images.

To demonstrate the capabilities of the system, we systematically evaluated and categorized the dynamic behavior of mitochondria and correlated these dynamics with mitochondrial morphology and mitochondrial protein distribution at the nanoscale level. Mitochondria are essential for the health of neuronal cells, and changes in mitochondrial dynamics as well as morphology have been shown to occur in neurodegenerative diseases such as Multiple Sclerosis, Parkinson's, Alzheimer's, Huntington's, Lou Gehrig's, and Schizophrenia [14]–[16]. However, identification of mitochondrial changes can be potentially confounded by the natural variability in morphology, dynamics, presence of mutations in mitochondrial DNA, maintenance of membrane potential, and even nanoscale distribution of membrane proteins [16]–[22]. The ability to characterize and correlate the dynamics and ultrastructure of a large number of organelles has the potential to allow detailed screening of disease pathologies.

## Results

**Figure 1:**
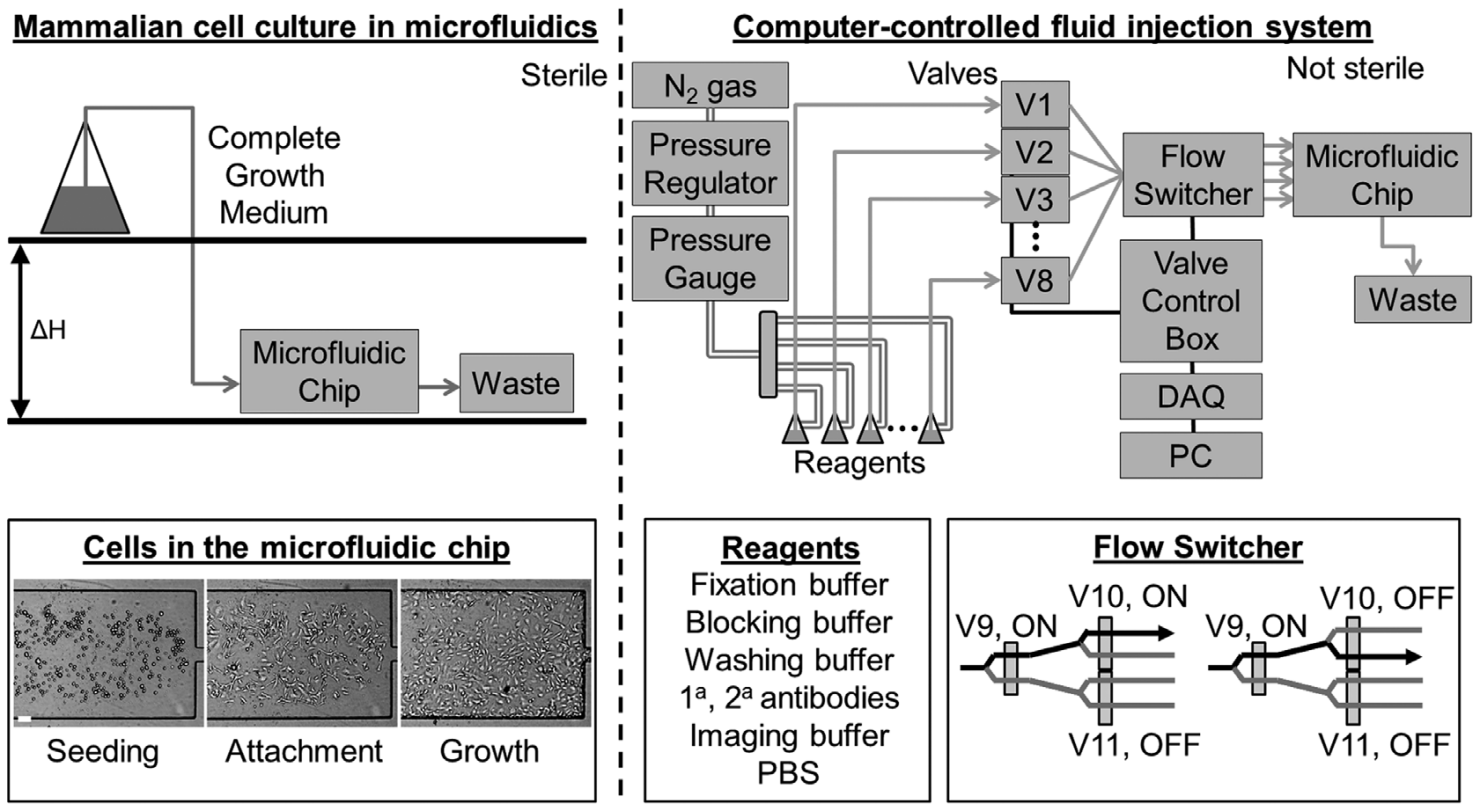
Design of microfluidic platform. Left, top: The cell culture is performed under sterile conditions. The flow is established using gravity-driven flow and the flow rate is adjusted by changing the value of *ΔH.* Left bottom: Cells are introduced into the microfluidic chip (seeding), allowed to attach (attachment), and then maintained under steady, continuous perfusion (growth). Right, top: During the data acquisition phase, the chip is removed from the sterile environment, placed on the microscope stage, and connected to a computer-controlled fluid delivery system. The number of reagents can be adjusted by adding or removing valves. Pressurized air is coupled to reagents which are routed through the system using solenoid pinch valves. Right, bottom: Each reagent can be delivered to each channel in the microfluidic chip through a binary multiplexer tree. By adjusting the configuration of the valves, fluid can be routed to different channels (shown are two examples). A total of *n-1* three-way solenoid valves are needed to split the incoming fluid from one channel to *n* channels.

There are a wide variety of microfluidic chip designs that have already been demonstrated for optical microscopy and/or immunostaining of bacteria and mammalian cells [12], [13], [23], many of which utilize a multi-layered design with on-chip valves [12], [24]. For simplicity and ease of use, we utilized a single-layered microfluidic chip manufactured using polydimethylsiloxane (PDMS) bonded to a glass coverslip (**Fig. 1** and **Fig. S1 in** **S1 File**; see Materials and Methods).

Direct implementation of established protocols in these devices for cell seeding, immunostaining, and STORM imaging buffers resulted in very low cell concentrations, poor labeling densities, and complete absence of the photoswitching of fluorophores that is necessary for STORM. These poor results could be attributed to two key factors. First, during cell seeding, the density of cells in suspension dramatically decreases when the diameter of the tube through which the cells flow is small, an effect that is commonly observed in blood cells flowing through capillaries, and referred to as the Fahraeus-Lindqvist effect [25]. This problem was overcome simply by increasing the cell seeding density by a factor of about 30. Second, most sample preparation protocols are optimized for the materials polystyrene and glass, which have significantly different material properties than PDMS [26]. The biggest challenge is that PDMS tends to absorb small molecules [27], thereby changing the local concentration of some (but not all) reagents in an unpredictable manner. We overcame these problems by systematically testing and determining the optimal concentration for the reagents used in immunostaining and STORM imaging buffer (see Materials and Methods).

**Figure 2:**
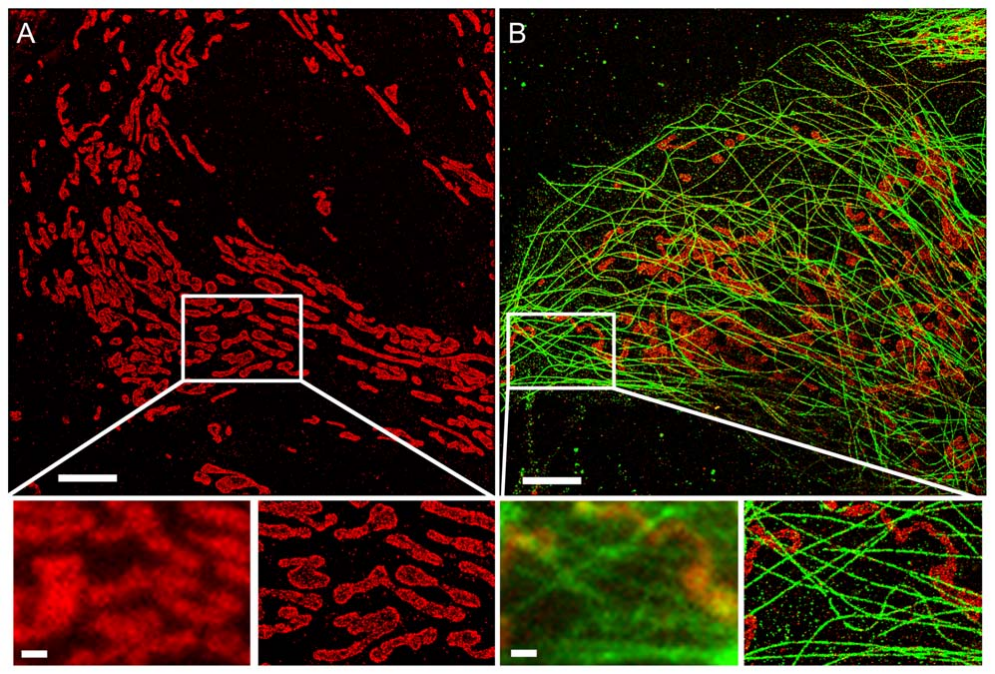
STORM microscopy in microfluidic chambers. Samples were prepared using a setup for automated immunostaining. (A) One color STORM image of mitochondria (Tom20). (B) Two-color STORM image of mitochondria (Tom20) and microtubules (alpha-tubulin). In both panels, the zoomed region shows the same region imaged using conventional epifluorescence microscopy (left) and STORM (right). Scale bars, 5 µm in top images, and 1 µm in bottom images (zoomed regions).

After optimizing mammalian cell culture, sample preparation, and imaging conditions, we were able to obtain labeling densities and imaging buffer conditions that allowed both single and multi-color super-resolution imaging with an image resolution of up to 23.8 nm (average image resolution of 37.5 nm) in the lateral directions (as determined from localization precision and Nyquist criterion for label density) (**Fig. 2** and **Fig. S2 in** **S1 File**). The combined system enabled fully-automated immunostaining. Samples prepared using the automated system showed no differences when compared to samples prepared manually (**Fig. 2**, prepared using the automated system, and **Fig. S2 in** **S1 File**, prepared manually).

**Figure 3:**
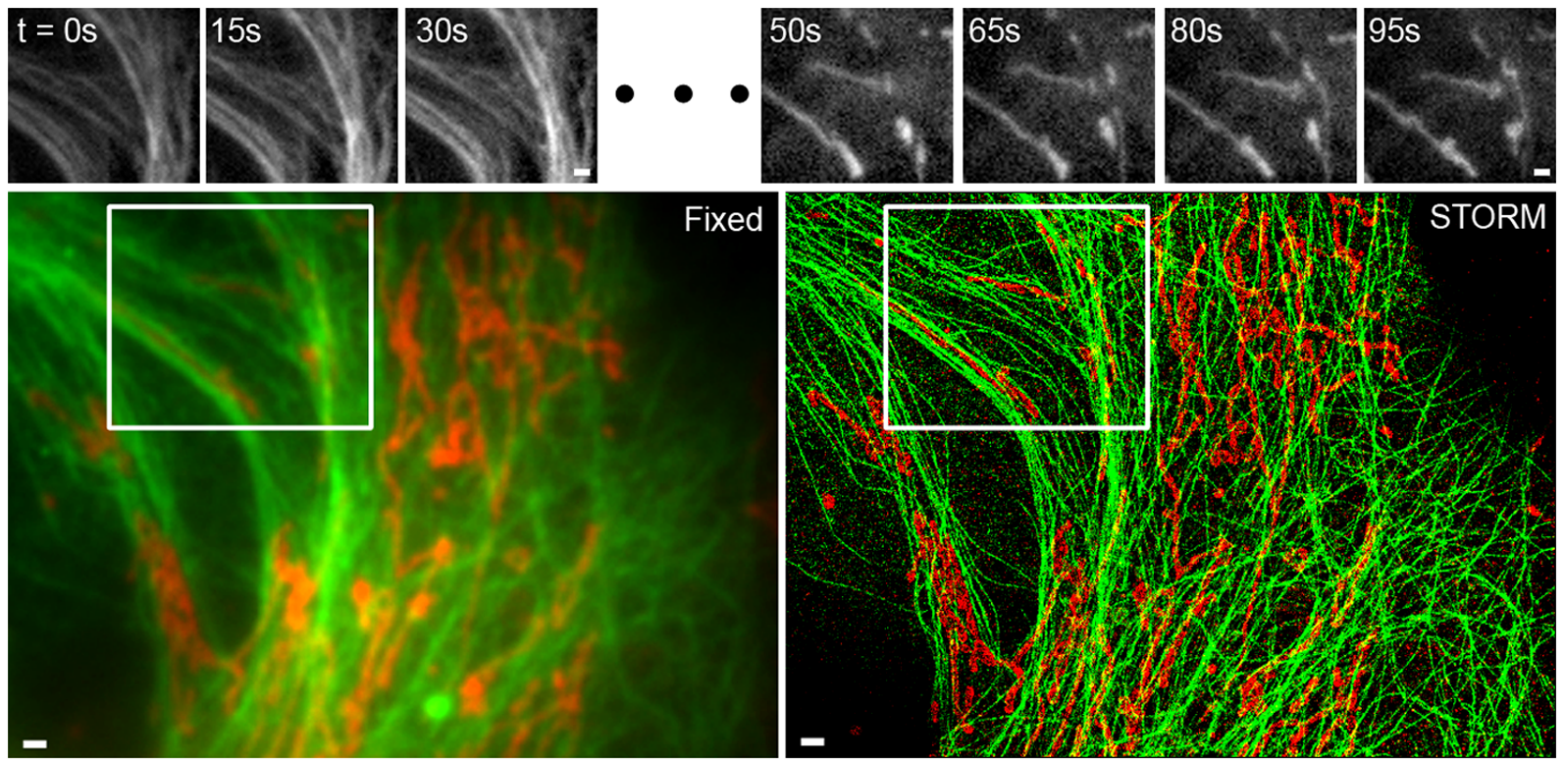
Correlative microscopy using microfluidics. Microtubules and mitochondria were imaged sequentially using epifluorescence microscopy and STORM. The live imaging examples from various time points selected from a sequence of live cell images acquired at 100 ms per frame (top) are from the region inside the white boxes (bottom). The experimental sequence was as follows: live-cell imaging of microtubules (top left), followed by live-cell imaging of mitochondria (top right), on-stage automated fixation, automated immunostaining of mitochondria, epifluorescence imaging of mitochondria (bottom left, red), STORM of mitochondria (bottom right, red), automated immunostaining of microtubules, epifluorescence imaging of microtubules (bottom left, green), and STORM imaging of microtubules (bottom right, green). Since the STORM images were acquired sequentially, fiduciary beads were used to align the two images. Scale bars, 1 µm.

By adding an additional fluid channel containing a fixative solution, to allow for computer-controlled on-stage fixation, we extended the system to perform correlated live-cell and STORM experiments in multiple colors based on a cross-talk-free sequential imaging approach [28] (**Fig. 3**). The automated system eliminated the most time-consuming, labor-intensive, and technically-challenging steps required for correlative live-cell and super-resolution microscopy and significantly reduced the amount of time needed for human supervision and manual input.

To validate the robustness and utility of the system, we applied this platform to investigate mitochondrial dynamics by correlating live mitochondrial dynamics with mitochondrial morphology and protein organization in STORM images (**Fig. S3 in** **S1 File** **,** **S1 Movie** **and** **S2 Movie**). We carried out a total of 28 correlative live-cell super-resolution experiments in a time period of about 65 hours with high quality data in 21 out of 28 experiments (75% success rate). In correlative microscopy, the cell imaged for time-lapse microscopy has to be the same cell imaged in STORM. Therefore, it is not possible to select the best labeled cell for STORM imaging, which is the common procedure when fixed cells alone are imaged. Since there is always a level of variability in immunostaining (regardless of the use of microfluidic devices), there are instances in which the cell selected for time-lapse imaging will have less than optimal labeling density for STORM. The experimental time we could achieve using the microfluidic devices was a significant improvement compared to manual throughput of one experiment per day. Most importantly, the immunostaining was carried out unsupervised.

The correlative imaging enabled us to investigate the relationships between dynamics, size, and protein distribution at the level of individual organelles for a large population of mitochondria. Even though mitochondrial dynamics (in particular mitochondrial fusion and fission) have previously been imaged using live-cell STORM at a temporal resolution of few seconds, one important advantage of the correlative approach is the much lower laser power densities used for live imaging (0.2 to 0.5 W/cm^2^ of 561-nm light was used as opposed to 10 kW/cm^2^ of 561-nm light used in live-cell STORM [7]). Given that mitochondria are especially fragile organelles which fragment easily due to photodamage [29], laser intensity is an important consideration. A second advantage of the correlative imaging approach is the fact that even with the extremely low laser power densities used; we could still probe rapid and transient dynamic processes that take place in millisecond time scales, which is not possible with live-cell STORM. Finally, no imaging buffer or oxygen scavenger system was required for the live cell imaging. Cells were maintained in complete growth medium during live cell imaging (see Materials and Methods). These considerations help to minimize any undesired perturbations to the cell or to the mitochondria.

**Figure 4:**
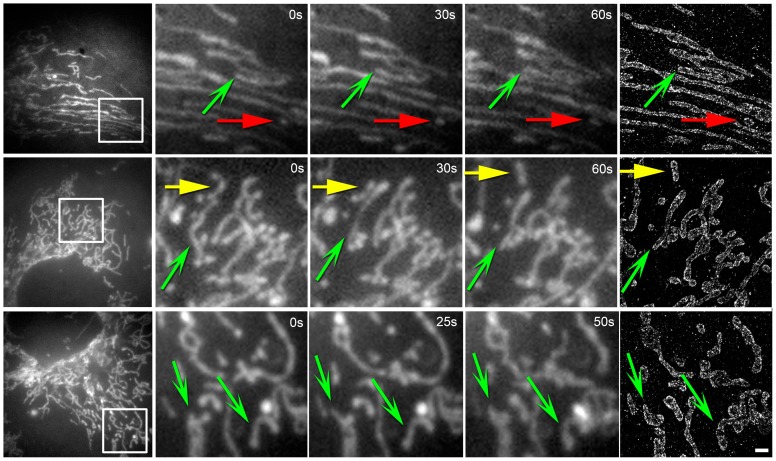
Examples of mitochondria events observed using correlative live-cell and super-resolution microscopy in a microfluidic device with a fluid delivery system for computer-controlled fixation and immunostaining. Each row represents one dataset, showing a region of interest within the cell at three different time points selected from a sequence of live cell images acquired at 50 ms per frame during live-cell imaging followed by the STORM image (from left to right). In each dataset, mitochondria were systematically identified and categorized based on their dynamic category using both the STORM image and the live-cell video (see S1 Movie). Examples of dynamic categories are labeled using color-coded arrows (red  =  static, green  =  dynamic-slow, yellow  =  dynamic-fast; sharp arrowhead  =  interacting, flat arrowhead  =  isolated). Scale bar, 1 µm.

We identified a total of 577 mitochondria (357 in wild type cells and 220 in cells stably expressing GFP-tubulin) (**Fig. 4** and **Fig. S3 in** **S1 File**, see Materials and Methods) that could be clearly discerned both in a live video showing mitochondrial dynamics up to the point of fixation, as well as in a high resolution STORM image. Here, it was important to utilize the STORM image to distinguish between closely-spaced mitochondria, to discern their size, and to examine the distribution of proteins along their membrane, details that were obscured due to the diffraction-limit in epifluorescence microscopy utilized for live-cell imaging. We first investigated the characteristics of mitochondrial dynamics. Each mitochondrion was assigned to one of three categories: stationary, undergoing a slow, but dynamic motion (dynamic-slow), or undergoing a fast translation across the cell (dynamic-fast) (**Fig. 4**). Stationary mitochondria appeared to be docked and single particle tracking showed that their motion was confined to a region around 192±91 nm (total displacement in the first 500 frames of the time lapse movie) (**Fig. S4A in** **S1 File** **and** **S3 Movie**). Dynamic-slow mitochondria appeared to shift back and forth and moved within a larger range of 414±261 nm in the same time period (p = 0.00038). While they did not show processive, directed translocation in the cell, often the mitochondrial morphology underwent dynamic changes (**Fig S4B in** **S1 File** **and** **S4 Movie**). Finally, dynamic-fast mitochondria were those organelles that underwent a net processive translocation (∼1570 nm on average) from one part of the cell to another part, reaching an average speed of ∼700 nm/sec during periods of processive motion, consistent with motor-protein mediated active transport along microtubules (**Fig S4C in** **S1 File** **and** **S5 Movie**). The majority of mitochondria underwent the dynamic-slow motion (83.5%), followed by a small fraction of stationary (10.5%) and dynamic-fast (5.9%) mitochondria.

To further characterize the types of interactions that occurred between mitochondria, we also determined whether each mitochondrion contacted other neighboring mitochondria (interacting), as opposed to remaining completely isolated from all other mitochondria (isolated). The majority of mitochondria were interacting (71.6%) as opposed to isolated (28.4%). It is possible that the low spatial resolution of conventional microscopy leads to an overestimation of the interacting category, since a fraction of the mitochondria that appear to overlap in conventional microscopy may actually not touch each other at the super-resolution level. Interestingly, the majority of interacting mitochondria were those that belonged to the dynamic-slow (**Table 1**) category. In contrast, both stationary and dynamic-fast mitochondria tended to be isolated.

**Table 1:** Distribution of mitochondrial dynamics.

	Stationary	Dynamic-slow	Dynamic-fast
Interacting	10 (2.8%)	241 (67.5%)	6 (1.7%)
Isolated	27 (7.6%)	61 (17.1%)	12 (3.4%)

**Figure 5:**
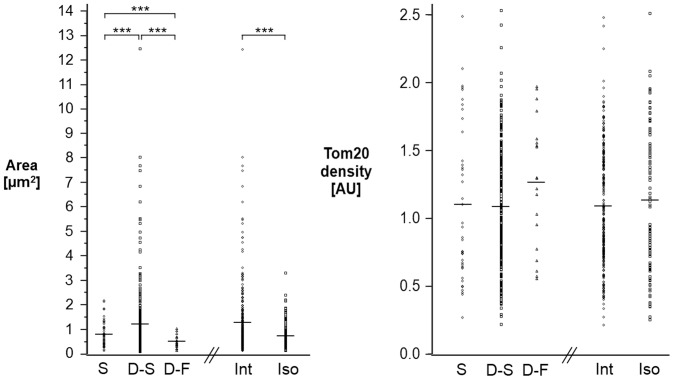
Mitochondrial dynamics in relation to size and protein distribution (S, Stationary, D-S, Dynamic-Slow, D-F, Dynamic-Fast; Int, Interacting, Iso, Isolated). The average is indicated by a horizontal line. Tom20 density is given as the number of Tom20 localizations per unit area normalized to the overall median density. All dynamic categories were significantly different from each other in area (***, p<0.001), but were not significantly different in Tom20 density.

Next, mitochondrial size determined from STORM images was correlated to mitochondrial dynamics determined from the live-cell videos. Mitochondria that were dynamic-slow or interacting were larger in size when compared to all other categories (p<0.001) (**Fig. 5**). Furthermore, dynamic-fast mitochondria were on average smaller than stationary mitochondria (p<0.001). Together, this data suggests that mitochondrial size, dynamics, and interactions are not independent parameters, but rather, related to each other.

We also examined whether the membrane distribution of mitochondrial protein Tom20 was related to mitochondrial dynamics. Protein distribution is not necessarily constant across all mitochondria and can depend on different factors. For example, a previous study found variations in Tom20 levels across mitochondria based on the position of these mitochondria relative to the nucleus [18]. Since Tom20 is an outer membrane protein, we wondered if its distribution is also sensitive to the shape changes experienced by the dynamic-slow or dynamic-fast mitochondria. Tom20 density (number of Tom20 localizations per unit area) showed a broad distribution within each dynamic category. There was not a statistically significant difference in the mean Tom20 density across the different dynamic categories (**Fig. 5**). This result indicates that the distribution of Tom20 is not sensitive to mitochondrial dynamics.

Finally, to demonstrate that this kind of streamlined and automated correlative imaging and analysis can be applied to screen changes in mitochondrial dynamics, morphology, and protein distribution we compared these parameters to cells stably expressing a GFP-tubulin marker. Since dynamic mitochondria are likely associated with microtubules, we wondered if over-expression of GFP-tagged tubulin could alter the correlations in the dynamic and static categories that we observed in the wild type cells. Overall, the distribution of mitochondrial dynamics was similar between wild-type and transfected cells (**Table S1 in** **S1 File**). Likewise, there were no statistically-significant differences when comparing mitochondrial morphology and protein distribution between dynamic categories (**Table S2 in** **S1 File**). This result confirmed that protein over-expression did not alter this particular organelle in these stably-transfected cells at the level of its intracellular dynamics, nanoscale morphology, and nanoscale Tom20 membrane distribution.

## Discussion

We have combined correlative super-resolution and live-cell imaging with PDMS-based microfluidic devices, automated and streamlined the sample preparation, and demonstrated an application to investigate mitochondrial dynamics in the context of their high-resolution morphology and their nanoscale membrane protein distribution (**Fig. 4** **,** **Fig. 5** **,** **Fig. S3 in** **S1 File**, **S1 Movie** **, and** **S2 Movie**). To the best of our knowledge, this is the first time that STORM has been successfully demonstrated using mammalian cells in microfluidic devices. Moreover, we show for the first time that correlative live cell and STORM can be performed using microfluidics.

From a practical perspective, the design we used is simple and low-cost, making it accessible to laboratories without previous expertise in microfluidics. The use of external valves allows the microfluidic chip to be easily transported on and off the microscope stage, an important advantage over microfluidic chips that utilize on-chip valves [12]. As a result of the external valves, the cell culture can be maintained off the microscope stage, and if needed the chip can be removed from the microscope stage when no imaging is being carried out and brought back at a later time for further imaging using a virtual grid approach to relocate the same region of interest [28]. This capability frees up the microscope during times where no data is collected, and allows for multiple batches to be prepared in parallel (**Fig. 1** **and Fig. S3 in** **S1 File**). Most designs that use on-chip valves do not allow this flexibility since the microfluidic device must typically be connected to a pressure source to keep the valves in their closed position. Selecting the simplest possible design minimizes operation cost and increases the reliability of the overall system. In particular, unlike previous systems, our imaging chamber is relatively large (2 cm×1 mm, see **Fig. S1 in** **S1 File**). The use of a large imaging chamber enables cells to grow in larger populations, as a nearly-confluent monolayer providing healthy growth conditions and giving the researcher the freedom to select an appropriate cell for imaging. This is an important consideration when working with transfection of cells, which typically results in nonuniform expression levels due to the random insertion of DNA – in this case it is important to be able to select a cell that has an appropriate expression level. Finally, the modular design makes the microfluidic device flexible and provides a platform on which many future designs and experiments can be built. Although we have elected to use a very simple microfluidic chip, more advanced microfluidic techniques can be introduced by swapping out our current chip and by replacing it with a new design. With our current design, we have the ability to rapidly change the cellular microenvironment with fast fluid exchange. However, by replacing the chip with a new design, it would also be possible to generate and apply microgradients in concentration or in temperature [30]-[32]. Precise control over the cellular microenvironment could give important insights into how cells modify protein distributions in response to different perturbations.

We have addressed a number of challenges that are associated with implementing microfluidics with other technologies, such as the additional optimization experiments that are needed to carry out immunostaining and STORM imaging. Sample preparation is especially important for high-resolution imaging methods such as STORM, since the improved resolution requires an even more stringent criterion for the preservation and labeling of sample features after fixation. In particular, high labeling densities must be achieved using higher-than-typical antibody concentrations. The labeling density is directly linked to the spatial resolution through the Nyquist criterion (the labeling density must be such that the distance between individual localizations in the resulting image is at least half of the desired resolution) [4], [33], [34]. In addition, the imaging buffer conditions are crucial for inducing the desired photoswitching properties in the small organic fluorophores. Our results establish that with properly optimized sample preparation protocols, microfluidics and STORM can be combined. As a general guideline for future implementations of PDMS-based microfluidic chambers for STORM, based on our optimization tests, the antibody concentrations should be increased by about five-fold. The buffer conditions determined here should in principle work for all photoswitchable fluorophores that have similar photoswitching properties in thiol-based buffers. Together, these important optimization steps form the basis for integrating microfluidics with STORM microscopy.

We have developed a tool that can assay mitochondrial dynamics at a high temporal resolution (up to 50 ms temporal resolution) in relation to their morphology and protein distribution (up to 23.8 nm spatial resolution). Our data suggests that there may be different functional categories of mitochondria within a cell and that the morphology and dynamic behavior of a mitochondrion may be linked. One potential limitation of this approach is that the imaging of mitochondrial dynamics is performed at a higher temporal resolution but lower spatial resolution, which may lead to two mitochondria appearing to "interact" while in fact remaining "isolated" when examining only the live-cell imaging data. This miscategorization will lead to an overestimation of the interacting category. While there are currently other techniques available for live-cell imaging at higher spatial resolutions, the advantage of our approach is that we can achieve high temporal resolution (∼50 ms) at a very low laser power density (∼0.2 W/cm^2^ at 561-nm) which minimizes and potential disturbances to the cell due to possible phototoxic effects.

In summary, we have demonstrated a versatile platform based on microfluidics for automated correlative microscopy that has the capability to image with both high temporal resolution (live-cell) and high spatial resolution (STORM). This platform adds to a growing list of recent “systems microscopy” approaches [35]–[38] that address the need for automation in advanced microscopy and can be applied to investigate biological phenomena at very high spatial and temporal scales with a wide range of potential applications which include cargo transport [11] and mitochondrial motility.

## Materials and Methods

### Microfluidic Chip Design and Fabrication

Microfluidic chips with miniaturized imaging chambers were designed using AutoCAD (Autodesk, Inc., Sausalito, CA) and fabricated by the Stanford Microfluidics Foundry in PDMS (RTV615, General Electric) using soft lithography. A single layer of PDMS was plasma bonded to a glass coverslip (Gold Seal Cover Slips, Thomas Scientific, Swedesboro, NJ). Each chip contains eight independent channels with a uniform flow height of 100 µm, a key difference with previously used on-chip designs for immunostaining of mammalian cells [12]. A height of 100 µm allows for approximately four hours of culture time without fluid exchange [39] (important to allow for cell attachment, **Fig. 1**). Since sterile conditions are critical for mammalian cell culture, but since the maintenance of sterility throughout an experiment can add unnecessary complexity and cost, we defined two working zones (**Fig. 1**). In the first zone, adherent mammalian cells are introduced and grown in a microfluidic chip under sterile conditions, using a conventional incubator. In the second zone, for the data collection portion of correlative imaging, since sterility was not necessary, the microfluidic device was transferred to the microscope for imaging once cells have adhered and reached the proper level of confluency.

### Automated Fluid Delivery System

The number of reagents and the timed sequence in which they are delivered varied depending on the immunostaining protocol and on the experimental conditions. We therefore developed a multiplexed design that could be easily modified to give flexibility to experimental strategy (**Fig. 1**). Instead of assigning each imaging channel to its own dedicated set of reagents, all imaging channels have access to a common set of fluid reservoirs. This allowed the numbers of reagents or imaging channels to be increased or decreased independently of upstream or downstream components, an important advantage over more complex and less flexible former designs that use on-chip valves [12]. Reagents were loaded into either 15 mL centrifuge tubes or Tygon microbore tubing (0.020″×0.060″ inner and outer diameter, Cole Parmer, Vernon Hills, IL), connected to a Luer manifold, and pressurized with filtered Nitrogen gas. Fluid delivery was regulated using external solenoid pinch valves (2-way normally closed pinch valves and 3-way pinch valves outfitted for 0.023″×0.093″ inner and outer diameter C-Flex tubing, Bio-Chem Fluidics, Boonton, NJ). A data acquisition card (DAQ) (USB 6501, National Instruments) in combination with custom-written software (LabVIEW, National Instruments) and a custom-built electronics box was used to drive the solenoid valves. The DAQ configuration allowed for up to 24 digital outputs, organized into three ports of eight outputs. Instead of using each output to control one valve (resulting in a maximum of 24 valves), the first two ports were multiplexed to allow for more than 2,000 valves to be addressed by the DAQ (utilizing the first port to select which set of eight valves to control, and the second port to actuate up to eight valves). DAQ signals were read by a custom-built electronics box and used to switch individual power circuits on/off to provide the appropriate power needed for controlling the solenoid valves (24VDC at 150 mA per valve). The custom-built electronics box consisted of a rack-mount design with individual modules, each of which controlled up to eight valves. Together, the DAQ multiplexing and the rack-mount design provided a simple way to scale up or down the total number of solenoid valves. To minimize heating of solenoid valves, after 100 ms the input voltage was reduced to 8VDC, which still enabled the valves to remain on (Coolcube, Biochem Fluidics, Boonton, NJ). The LabView software to run the microfluidic device, along with the documentation on how to install and run the software can be found at https://github.com/LakadamyaliLab/microfluidic-control/blob/master/Automated_Valve_Control.zip

### Optimization of Fluid Delivery in the Automated Immunostaining System

During microscopy, the pressure could be increased to generate rapid fluid exchange (**Fig. 1**). Using pressure-driven flow enabled precise control over fluid flow. We performed calibration experiments to establish the appropriate flow rates needed for on-stage fluid delivery (**Fig. S5 in** **S1 File**). Two-way and three-way solenoid pinch valves (075P2NC24-23B and 075P3MP24-23B, Biochem Fluidics, Boonton, NJ) with zero dead volume in combination with microbore tubing (Tygon microbore tubing, 0.020″×0.060″ inner and outer diameter, Cole Parmer, Vernon Hills, IL, and C-Flex tubing, 0.023″×0.093″ inner and outer diameter, Biochem Fluidics, Boonton, NJ) were used to minimize reagent consumption. Although the pinch valves were placed as close to the stage as possible to minimize the travel distance between the reagent reservoirs and the microfluidic chip, there was still a residual distance of 7′ of Tygon microbore tubing between the imaging chamber in the microfluidic chip and the solenoid pinch valves, corresponding to a fluid overhead of approximately 35.5 µL. In addition, the binary multiplexer tree (for routing fluids between the different channels) required an additional 33.4 µL, which would have resulted in a combined fluid overhead of 68.9 µL per channel. We were able to decrease this overhead down to 20 µL per channel by using an alternate fluid delivery strategy based on moving a smaller bolus of fluid (e.g. having a bolus of antibody solution sandwiched by different fluids upstream and downstream). To ensure that the bolus was delivered to the chip, we tuned the fluid delivery such that the bolus was present both upstream and downstream of the chip. This corresponded to an overall reagent usage of 75 µL per channel (which includes a fluid overhead of 20 µL), or 300 µL per experiment (with four channels).

### Mammalian Cell Culture

Prior to cell seeding, microfluidic chips and components were sterilized. Components were either purchased sterile, autoclaved, or sterilized using a combination of ethanol and ultraviolet light, as described below. For the sterilization step, the complete cell culture system was assembled in a sterile hood and a gravity-driven flow of pure ethanol was established. Care was taken during the initial priming steps (that is, during the initial filling of the tubing upstream of the microfluidic chip) to eliminate any bubbles. After priming, introduction of ethanol into the dry microfluidic chip by means of gravity flow resulted in complete filling of the microfluidic chip without bubbles. Once the gravity flow was established, the system was exposed to ultraviolet light for at least thirty minutes. Next, the ethanol was carefully replaced with complete growth medium, and gravity flow was re-established. Care was taken to not introduce any bubbles into the system. The complete growth medium was allowed to flow through the system overnight to equilibrate the PDMS with the complete growth medium. African green monkey kidney cells (BS-C-1, American Type Culture Collection, ATCC CCL-26) were seeded into microfluidic chambers at a seeding density of 4 to 6 million cells per mL and placed into an incubator and maintained at 37°C and 5% CO_2_ for 2.5 to 4.5 hours to allow cells to attach to the bottom of the chamber. After attachment, slow but steady perfusion of complete growth medium (CGM) was established (Minimum Essential Medium, with Earle's salts and nonessential amino acids plus 10% (v/v) FBS, 2 mM L-glutamine, 1 mM sodium pyruvate, and a mixture of penicillin streptomycin; complete growth medium components purchased from GIBCO, Life Technologies). We performed calibration experiments to establish the appropriate flow rates needed for cell culture (**Fig. S1 and S5 in** **S1 File**). This slow, steady flow rate was established using gravity-driven flow controlled by changing the difference in height between the media and waste reservoirs, without the need for any electrical or mechanical pumps or parts. For two-color imaging, immediately prior to cell seeding, microfluidic chips were incubated with fiduciary beads (Nile Red, Spherotech), which were used to precisely align the sequentially-acquired STORM images of mitochondria and microtubules.

### Live-cell Imaging

Live-cell imaging was performed using a previously-described custom-built microscope [11]. Briefly, cells were imaged in epifluorescence wide-field microscopy using a 100×1.4 NA oil-immersion objective. Cells were maintained on-stage at 37°C using a temperature-controlled objective and stage heater (Live Cell Instrument). For one-color mitochondrial imaging, cells were incubated with MitoTracker orange (Invitrogen) at a concentration of 1 µM in CGM for 10 minutes at 37°C, followed by a washing step of CGM. Cells were maintained in CGM during the live-cell imaging. A 560-nm fiber laser (MPB Communication) was used to excite the MitoTracker at a power between 0.2 and 0.5 W/cm^2^. The emitted fluorescence passed through an emission filter (ET605/52, Chroma) and was collected with an electron-multiplying charge-coupled device (EM-CCD, Andor Technology) at a frame rate of either 10 or 20 frames per second. For two-color imaging, the same procedures were performed with the following additional steps. During cell seeding, a well-established stably-transfected cell line for GFP-tubulin was used, which were generated by transfecting BS-C-1 cells with a plasmid encoding GFP-tubulin and selectively screened through multiple passages (plasmid kind gift of Lynne Cassimeris, Lehigh University, Bethlehem, PA). A 488-nm line from an argon-krypton laser (Spectrum IC70, Coherent) was used to excite GFP at 1.0 W/cm^2^ and emitted fluorescence was collected after passing through an emission filter (ET525/50, Chroma).

### Automated Immunostaining

Cells were fixed using the computer-controlled fluid delivery system with 3% (w/v) paraformaldehyde and 0.1% (w/v) gluteraldehyde diluted in PBS for 20 to 60 minutes, and then washed with PBS. Immunostaining was performed using the automated fluid delivery system. Microfluidic chambers were stained using a synchronized routine (e.g. for each step, fluid was delivered to channel 1, 2, 3, and then 4, followed by the appropriate incubation times). For the mitochondrial labeling, cells were incubated with blocking buffer for 60 minutes at room temperature (3% (w/v) BSA and 0.2% (v/v) TritonX-100 in PBS, with a fresh injection of blocking buffer every 10 minutes), followed by primary antibody in blocking buffer for 50 minutes (Tom20, sc-11415, Santa Cruz; dilution 1∶10), washing buffer for 10 minutes (0.2% BSA, 0.05% TritonX-100, with a fresh injection of washing buffer every 5 minutes), secondary antibody in blocking buffer for 40 minutes (Affinity Pure Donkey Anti Rabbit IgG (H+L) 711-025-152, Jackson ImmunoResearch; custom-labeled in-house with Alexa Fluor 405 Carboxylic Acid Succinimidyl Ester (A30000, Invitrogen) and Alexa Fluor 647 Carboxylic Acid Succinimidyl Ester (A20006, Invitrogen) as described previously [40]), and a washing step (a one-time injection of washing buffer followed immediately by a one-time injection of PBS). The microtubule staining protocol was identical to the one used to stain mitochondria, except for the following changes: for blocking buffer, an incubation time of 5 minutes was used, for primary antibody, rat monoclonal (YL1/2) to α-tubulin (ab6160, Abcam) was used at a dilution of 1∶30, and for secondary antibody, donkey anti-rat at a dilution of 1∶10 was used (Affinity Pure Donkey Anti Rat IgG (H+L) 712-005-150).

### STORM Imaging

Immediately prior to STORM imaging, an imaging buffer was injected into the microfluidic channel that was being imaged. The imaging buffer was made fresh by mixing an oxygen scavenger solution, a thiol solution, and a solution of 50% glucose in water at a ratio of 1∶40∶40. The oxygen scavenger solution was prepared by combining 14 mg glucose oxidase (Sigma Aldrich), 200 µL dilution buffer (10 mM Tris and 50 mM NaCl), and 50 µL of catalase (20 mg/mL, Sigma Aldrich), centrifuged for 1 minute. The thiol solution was prepared by dissolving 66 mg of cysteamine (Sigma Aldrich) in 360 mM HCl. STORM data acquisition was performed as described previously [11].

### STORM data analysis

Raw STORM data was analyzed using custom-written software (Insight3, kindly provided by Bo Huang, University of California, San Francisco). Insight3 was used to detect and localize single molecules in the raw STORM data, to correct for any sample drift, and to render a high-resolution image of localized molecules. Briefly, detection was performed based on a threshold and localization was performed by fitting detected points to a simple Gaussian to determine the *x* and *y* positions. Drift was calculated by splitting the raw data into 500–1000 frame subsets, reconstructing STORM images on these subsets, and correlating the resulting images to a reference image (the STORM image from the first subset). The final, drift-corrected locations of each molecule were saved in a separate file for additional analysis. For the multi-color datasets, fiduciary beads were used as previously described to precisely align the sequentially-acquired STORM images [11]. Briefly, fiduciary beads, which were visible alongside the blinking fluorophores in the raw STORM data, were detected, localized, corrected for drift, and rendered alongside the STORM images of mitochondria and microtubules using Insight3. Fiduciary beads from each color were then localized and compared to calculate the shift between the two images. The images were then shifted with respect to each other. This enabled precise alignment.

### Classification of mitochondrial dynamics

Mitochondria were systematically identified and classified according to their dynamic category using custom-written software in addition to ImageJ. Only those mitochondria which were clearly identifiable in both the high-resolution STORM image and its corresponding live video were included in the analysis. Specifically, those mitochondria which were partially out of focus, residing in dense regions with overlapping mitochondria, or exhibiting multiple dynamic behaviors were excluded from analysis. Based on examination of the live videos, mitochondria were manually classified as isolated, interacting, dynamic-slow, dynamic-fast, and stationary as defined in the manuscript. All classifications were performed by one trained operator who was blind to quantitative data generated from the STORM images. In addition, a particle and filament tracking program (FIESTA) [41] was used to confirm the classification based on the maximum displacement of mitochondria from their starting position as well as the average speed. Dynamic slow and static mitochondria had similar average speeds (∼310 nm/s), but could be distinguished based upon whether or not they were confined to a region within the cell. Static mitochondria were confined within a smaller region (total displacement  = 192±91 nm determined from the first 500 frames of the time lapse movie) compared to the dynamic slow mitochondria (total displacement  = 414±261 nm determined from the first 500 frames of the time lapse movie, p = 0.00038). Dynamic fast mitochondria underwent processive, directed motion and reached an average speed of 700 nm/sec during these periods.

### Quantification of Tom20 labeling density and image resolution

For each of the mitochondrion selected for analysis, an outline of the mitochondrion in the STORM image was manually generated using ImageJ. This outline was used to quantify the area in pixels^2^, which was then converted to µm^2^ based on the pixel size of the camera in the sample plane (0.157 µm/pixel in each direction). Next, using custom-written software, all the molecules which were localized inside the outline were selected and counted. The Tom20 labeling density was calculated by dividing the number of localizations within each mitochondria by the area of the mitochondria. Finally, the Tom20 density was normalized by dividing by the median density of the dataset. This approach in principle overestimates the Tom20 density since each fluorophore can give rise to more than one localization and each Tom20 may be labeled by more than one antibody. However, we assume that the density will be similarly overestimated across all dynamic categories, therefore not influencing the comparative analysis. The resolution (*R*) of the image was calculated by adding in quadrature a labeling-density-based estimate of image resolution (*R_1_*) and the localization precision of the microscope system (*R_2_*)[4], where
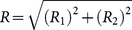


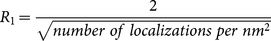


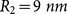



This calculation was performed for each mitochondria and then averaged to determine the average image resolution.

### Statistical Analysis

Groups were compared using two-tailed, unpooled *t*-tests with a significance value of 0.001.

## Supporting Information

S1 File **This file contains additional figures and tables.** Table S1, Distribution of mitochondrial dynamics in cells with GFP-tagged tubulin. Table S2, Comparison of mitochondrial morphology and protein distribution in stably-transfected cells vs. wildtype cells. Figure S1, Design of the microfluidic channels. Figure S2, Microfluidics is compatible with STORM. Figure S3, Overview of workflow. Figure S4, Representative trajectories for the static, dynamic slow, and dynamic fast categories of mitochondria. Figure S5, Calibration of the fluid delivery system.
(PDF)

S1 Movie **Example of a microfluidics-based correlative live-cell and super-resolution dataset.** In this video, the mitochondria are imaged live for 45 seconds and then fixed using a computer-controlled fluid delivery system (video sped up 5X). After automated immunostaining, the same region is imaged using STORM.
(AVI)

S2 Movie **Example of individual mitochondria from a microfluidics-based correlative livecell and super-resolution experiment.** In this video, mitochondria are imaged live for 30 seconds and then fixed using a computer-controlled fluid delivery system (left panel, video sped up 5X). After automated immunostaining, the same mitochondria are imaged using STORM (right panel).
(AVI)

S3 Movie **Representative example of a static mitochondrion (center of the frame) corresponding to the trajectory shown in Figure S4A in** **S1 File** **.** The video has been sped up 8X.
(AVI)

S4 Movie **Representative example of a dynamic-slow mitochondrion (center of the frame) corresponding to the trajectory shown in Figure S4B in** **S1 File** **.** The video has been sped up 8X.
(AVI)

S5 Movie **Representative example of a dynamic-fast mitochondrion (center of the frame) corresponding to the trajectory shown in Figure S4C in** **S1 File** **.** The video has been sped up 8X.
(AVI)

## References

[1] Rust MJ, Bates M, Zhuang X (2006) Sub-diffraction-limit imaging by stochastic optical reconstruction microscopy (STORM). Nat Methods 3:793–795. 16896339 10.1038/nmeth929 PMC2700296

[2] Betzig E, Patterson GH, Sougrat R, Lindwasser OW, Olenych S, et al (2006) Imaging intracellular fluorescent proteins at nanometer resolution. Science 313:1642–1645. 16902090 10.1126/science.1127344

[3] Hess ST, Girirajan TP, Mason MD (2006) Ultra-high resolution imaging by fluorescence photoactivation localization microscopy. Biophys J 91:4258–4272. 16980368 10.1529/biophysj.106.091116 PMC1635685

[4] Lakadamyali M, Babcock H, Bates M, Zhuang X, Lichtman J (2012) 3D multicolor super-resolution imaging offers improved accuracy in neuron tracing. PLoS One 7:e30826. 22292051 10.1371/journal.pone.0030826 PMC3265519

[5] Xu K, Zhong G, Zhuang X (2013) Actin, spectrin, and associated proteins form a periodic cytoskeletal structure in axons. Science 339:452–456. 23239625 10.1126/science.1232251 PMC3815867

[6] Huang B, Jones SA, Brandenburg B, Zhuang X (2008) Whole-cell 3D STORM reveals interactions between cellular structures with nanometer-scale resolution. Nat Methods 5:1047–1052. 19029906 10.1038/nmeth.1274 PMC2596623

[7] Shim SH, Xia C, Zhong G, Babcock HP, Vaughan JC, et al (2012) Super-resolution fluorescence imaging of organelles in live cells with photoswitchable membrane probes. Proc Natl Acad Sci U S A 109:13978–13983. 22891300 10.1073/pnas.1201882109 PMC3435176

[8] Jones SA, Shim SH, He J, Zhuang X (2011) Fast, three-dimensional super-resolution imaging of live cells. Nat Methods 8:499–508. 21552254 10.1038/nmeth.1605 PMC3137767

[9] Zhu L, Zhang W, Elnatan D, Huang B (2012) Faster STORM using compressed sensing. Nat Methods 9:721–723. 22522657 10.1038/nmeth.1978 PMC3477591

[10] Lakadamyali M (2013) Super-Resolution Microscopy: Going Live and Going Fast. Chemphyschem. 10.1002/cphc.201300720 24166886

[11] Balint S, Verdeny Vilanova I, Sandoval Alvarez A, Lakadamyali M (2013) Correlative live-cell and superresolution microscopy reveals cargo transport dynamics at microtubule intersections. Proc Natl Acad Sci U S A 110:3375–3380. 23401534 10.1073/pnas.1219206110 PMC3587250

[12] Cheong R, Wang CJ, Levchenko A (2009) High content cell screening in a microfluidic device. Mol Cell Proteomics 8:433–442. 18953019 10.1074/mcp.M800291-MCP200 PMC2649807

[13] Whitesides GM (2006) The origins and the future of microfluidics. Nature 442:368–373. 16871203 10.1038/nature05058

[14] Lovas JR, Wang X (2013) The meaning of mitochondrial movement to a neuron's life. Biochim Biophys Acta 1833:184–194. 22548961 10.1016/j.bbamcr.2012.04.007 PMC3413748

[15] Chen H, Chan DC (2009) Mitochondrial dynamics—fusion, fission, movement, and mitophagy—in neurodegenerative diseases. Hum Mol Genet 18:R169–176. 19808793 10.1093/hmg/ddp326 PMC2758711

[16] Nikic I, Merkler D, Sorbara C, Brinkoetter M, Kreutzfeldt M, et al (2011) A reversible form of axon damage in experimental autoimmune encephalomyelitis and multiple sclerosis. Nat Med 17:495–499. 21441916 10.1038/nm.2324

[17] Collins TJ, Berridge MJ, Lipp P, Bootman MD (2002) Mitochondria are morphologically and functionally heterogeneous within cells. EMBO J 21:1616–1627. 11927546 10.1093/emboj/21.7.1616 PMC125942

[18] Wurm CA, Neumann D, Lauterbach MA, Harke B, Egner A, et al (2011) Nanoscale distribution of mitochondrial import receptor Tom20 is adjusted to cellular conditions and exhibits an inner-cellular gradient. Proc Natl Acad Sci U S A 108:13546–13551. 21799113 10.1073/pnas.1107553108 PMC3158204

[19] Chan DC (2006) Mitochondria: dynamic organelles in disease, aging, and development. Cell 125:1241–1252. 16814712 10.1016/j.cell.2006.06.010

[20] Detmer SA, Chan DC (2007) Functions and dysfunctions of mitochondrial dynamics. Nat Rev Mol Cell Biol 8:870–879. 17928812 10.1038/nrm2275

[21] Plucinska G, Paquet D, Hruscha A, Godinho L, Haass C, et al (2012) In vivo imaging of disease-related mitochondrial dynamics in a vertebrate model system. J Neurosci 32:16203–16212. 23152604 10.1523/JNEUROSCI.1327-12.2012 PMC6794024

[22] Jans DC, Wurm CA, Riedel D, Wenzel D, Stagge F, et al (2013) STED super-resolution microscopy reveals an array of MINOS clusters along human mitochondria. Proc Natl Acad Sci U S A 110:8936–8941. 23676277 10.1073/pnas.1301820110 PMC3670330

[23] Cattoni DI, Fiche JB, Valeri A, Mignot T, Nollmann M (2013) Super-resolution imaging of bacteria in a microfluidics device. PLoS One 8:e76268. 24146850 10.1371/journal.pone.0076268 PMC3797773

[24] Unger MA, Chou HP, Thorsen T, Scherer A, Quake SR (2000) Monolithic microfabricated valves and pumps by multilayer soft lithography. Science 288:113–116. 10753110 10.1126/science.288.5463.113

[25] Fahraeus R, Lindqvist T (1931) The viscosity of the blood in narrow capillary tubes. American Journal of Physiology 96:562–568.

[26] Berthier E, Young EW, Beebe D (2012) Engineers are from PDMS-land, Biologists are from Polystyrenia. Lab Chip 12:1224–1237. 22318426 10.1039/c2lc20982a

[27] Toepke MW, Beebe DJ (2006) PDMS absorption of small molecules and consequences in microfluidic applications. Lab Chip 6:1484–1486. 17203151 10.1039/b612140c

[28] Tam J, Cordier GA, Borbely JS, Sandoval Alvarez A, Lakadamyali M (2014) Cross-Talk-Free Multi-Color STORM Imaging Using a Single Fluorophore. PLoS One 9:e101772. 25000286 10.1371/journal.pone.0101772 PMC4084994

[29] Mitra K, Lippincott-Schwartz J (2010) Analysis of mitochondrial dynamics and functions using imaging approaches. Curr Protoc Cell Biol Chapter 4: Unit 4 25 21–21. 10.1002/0471143030.cb0425s46 PMC3007120 20235105

[30] Jeon NL, Dertinger SKW, Chiu DT, Choi IS, Stroock AD, et al (2000) Generation of solution and surface gradients using microfluidic systems. Langmuir 16:8311–8316.

[31] Weibel DB, Whitesides GM (2006) Applications of microfluidics in chemical biology. Curr Opin Chem Biol 10:584–591. 17056296 10.1016/j.cbpa.2006.10.016

[32] Mao H, Yang T, Cremer PS (2002) A microfluidic device with a linear temperature gradient for parallel and combinatorial measurements. J Am Chem Soc 124:4432–4435. 11960472 10.1021/ja017625x

[33] Shroff H, Galbraith CG, Galbraith JA, Betzig E (2008) Live-cell photoactivated localization microscopy of nanoscale adhesion dynamics. Nat Methods 5:417–423. 18408726 10.1038/nmeth.1202 PMC5225950

[34] Dempsey GT, Vaughan JC, Chen KH, Bates M, Zhuang X (2011) Evaluation of fluorophores for optimal performance in localization-based super-resolution imaging. Nat Methods 8:1027–1036. 22056676 10.1038/nmeth.1768 PMC3272503

[35] Conrad C, Gerlich DW (2010) Automated microscopy for high-content RNAi screening. J Cell Biol 188:453–461. 20176920 10.1083/jcb.200910105 PMC2828931

[36] Conrad C, Wunsche A, Tan TH, Bulkescher J, Sieckmann F, et al (2011) Micropilot: automation of fluorescence microscopy-based imaging for systems biology. Nat Methods 8:246–249. 21258339 10.1038/nmeth.1558 PMC3086017

[37] Lock JG, Stromblad S (2010) Systems microscopy: an emerging strategy for the life sciences. Exp Cell Res 316:1438–1444. 20381488 10.1016/j.yexcr.2010.04.001

[38] Neumann B, Walter T, Heriche JK, Bulkescher J, Erfle H, et al (2010) Phenotypic profiling of the human genome by time-lapse microscopy reveals cell division genes. Nature 464:721–727. 20360735 10.1038/nature08869 PMC3108885

[39] Young EW, Beebe DJ (2010) Fundamentals of microfluidic cell culture in controlled microenvironments. Chem Soc Rev 39:1036–1048. 20179823 10.1039/b909900j PMC2967183

[40] Bates M, Huang B, Dempsey GT, Zhuang X (2007) Multicolor super-resolution imaging with photo-switchable fluorescent probes. Science 317:1749–1753. 17702910 10.1126/science.1146598 PMC2633025

[41] Ruhnow F, Zwicker D, Diez S (2011) Tracking single particles and elongated filaments with nanometer precision. Biophys J 100:2820–2828. 21641328 10.1016/j.bpj.2011.04.023 PMC3117161

