# Supplementary material for: A Microfluidic Platform for Correlative Live-Cell and Super-Resolution Microscopy
Source: PLoS One. 2014 Dec 29;9(12):e115512. doi: 10.1371/journal.pone.0115512 (PMC4278722; doi:10.1371/journal.pone.0115512)
Supplement: S1 File — This file contains additional figures and tables. Table S1, Distribution of mitochondrial dynamics in cells with GFP-tagged tubulin. Table S2, Comparison of mitochondrial morphology and protein distribution in stably-transfected cells vs. wildtype cells. Figure S1, Design of the microfluidic channels. Figure S2, Microfluidics is compatible with STORM. Figure S3, Overview of workflow. Figure S4, Representative trajectories for the static, dynamic slow, and dynamic fast categories of mitochondria. Figure S5, Calibration of the fluid delivery system. (PDF) [file pone.0115512.s001.pdf]

# Supporting Information

## Supporting Tables

### Table S1 – Distribution of mitochondrial dynamics in cells with GFP-tagged tubulin

| | Stationary | Dynamic-slow | Dynamic-fast |
|---|---|---|---|
| Interacting | 8 (3.6%) | 134 (60.9%) | 6 (2.7%) |
| Isolated | 11 (5.0%) | 52 (23.6%) | 9 (4.1%) |

The distribution of mitochondria across the dynamic categories is listed in stably-transfected cells expressing GFP-tagged tubulin (number of mitochondria, followed by percentage of the total number of mitochondria). The distribution of mitochondria across dynamic categories were similar between wild-type and transfected cells (see Table 1).

**Table S2 -** Comparison of mitochondrial morphology and protein distribution in stably-transfected cells vs. wildtype cells.

| Parameter | Dynamic Category | Wild type | Transfected | p-value |
|---|---|---|---|---|
| Area [$\mu m^2$] | S | 0.84 +/- 0.54 | 0.56 +/- 0.32 | 0.014 |
| | D-S | 1.23 +/- 1.40 | 1.07 +/- 1.53 | 0.244 |
| | D-F | 0.48 +/- 0.25 | 0.34 +/- 0.22 | 0.148 |
| Tom20 Density [AU] | S | 1.10 +/- 0.58 | 1.04 +/- 0.31 | 0.564 |
| | D-S | 1.09 +/- 0.44 | 1.06 +/- 0.39 | 0.473 |
| | D-F | 1.37 +/- 0.44 | 1.09 +/- 0.28 | 0.062 |

The morphology and protein distribution of mitochondria across different dynamic categories are not significantly different when comparing wild type cells to cells stably expressing GFP-tubulin.

## Supporting Figures

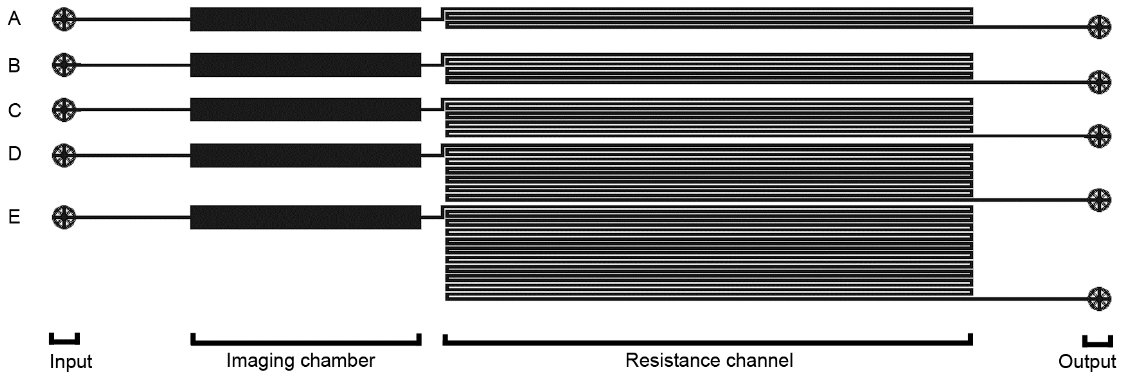


**Figure S1** – Design of the microfluidic channels. Each channel consists of an input, an imaging chamber, a long, folded resistance channel, and an output. The channel height was set to 10 µm. The imaging chamber had a width of 100 µm and a length of either 1 mm or 2 mm. The resistance channels had a width of 10 µm and lengths of 15.9, 20.7, 25.5, 35.0, and 54.1 cm (A, B, C, D, and E). Since each channel has a different flow rate under gravity flow conditions, initially, chips were manufactured to have all five of the channels (A, B, C, D, and E). After testing mammalian cell culture in these initial chips, it was found that channel A yielded the best results. Therefore, subsequent chips were manufactured with 8 redundant copies of channel A.

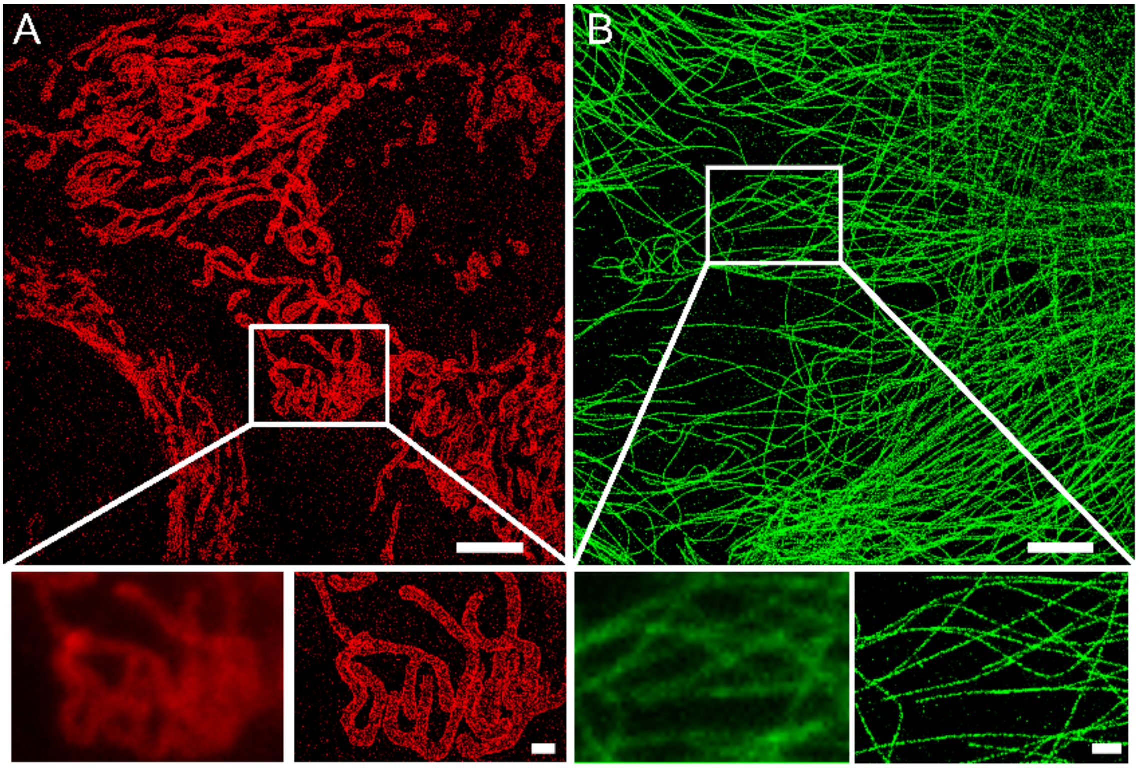


**Figure S2** – Microfluidics is compatible with STORM. Samples were prepared manually (manual fluid delivery). (A) STORM image of mitochondria (Tom20). (B) STORM image of microtubules (alpha-tubulin). In both panels, the zoomed region shows the same region imaged using conventional epifluorescence microscopy (left) and STORM (right). Scale bars, 5 µm in the top images, and 1 µm in the bottom images (zoomed regions).

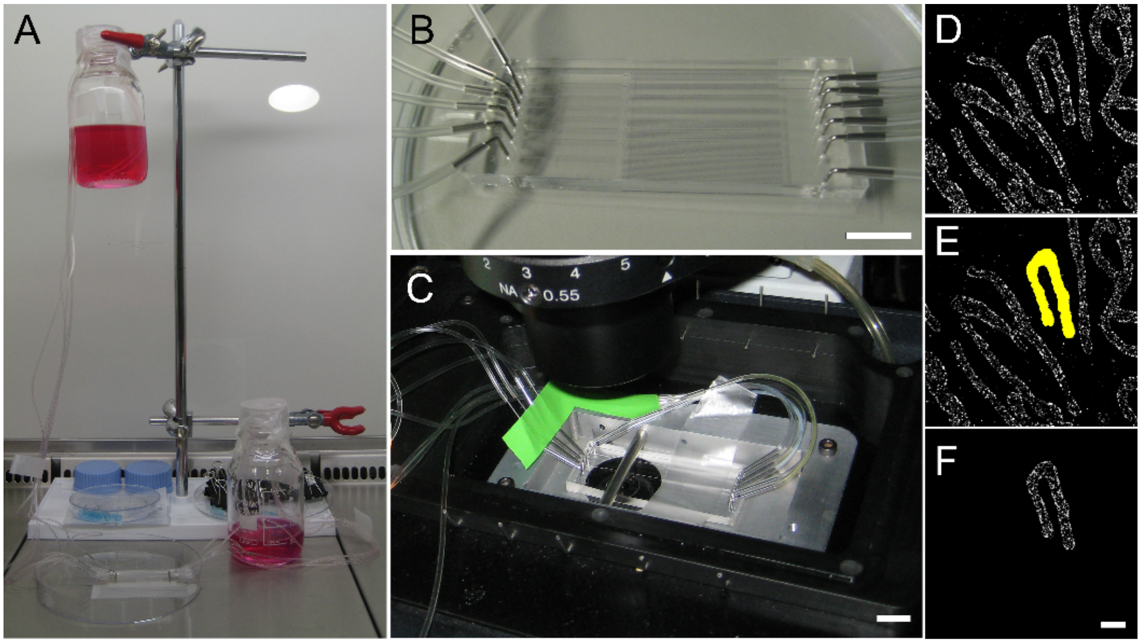


**Figure S3** – Overview of workflow. (A) Day 1. The microfluidic chip and cell culture materials are sterilized for thirty minutes and left overnight to equilibrate with complete growth medium under gravity flow, in a sterile hood. (B) Day 2. Mammalian cells are loaded into the microfluidic chip, left to attach for 2.5 to 4.5 hours (with no gravity flow), and then switched to perfusion culture overnight (with gravity flow). Scale bar, 1 cm. (C) Day 3. Live-cell imaging, on-stage fixation, immunostaining, and STORM imaging are performed in a sequential manner. Scale bar, 1 cm. (D-F) Day 4. (D) STORM datasets are processed by performing single molecule detection, localization, and rendering. (E) Individual mitochondria identified and selected for analysis using a combination of custom-written and publically-available software. Shown is an example of a single selected mitochondrion (yellow). (F) The localized molecules corresponding to the selected mitochondrion are stored for further analysis. Steps E and F are repeated for all mitochondria in the image satisfying the identification criteria. The microscope is occupied only on day 3. It is possible to combine days 3 and 4 by processing data as soon as it is recorded. Scale bar, 1 μm.

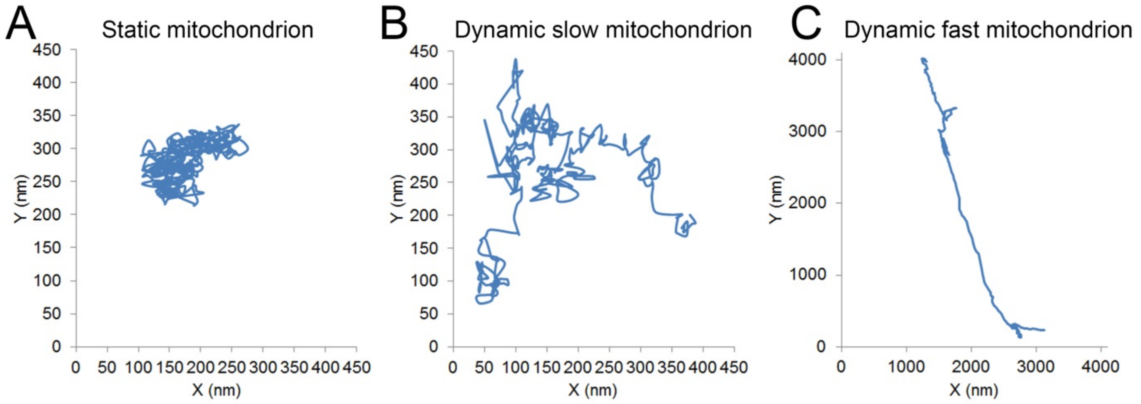


**Figure S4** – Representative trajectories for the static (A), dynamic slow, (B) and dynamic fast (C) categories of mitochondria. The trajectories of the static and dynamic slow mitochondria correspond to the first 500 frames (25 seconds) of the time-lapse movie. The trajectory of the dynamic fast mitochondrion corresponds to the period of directed, processive motion (293 frames, 14.65 seconds).

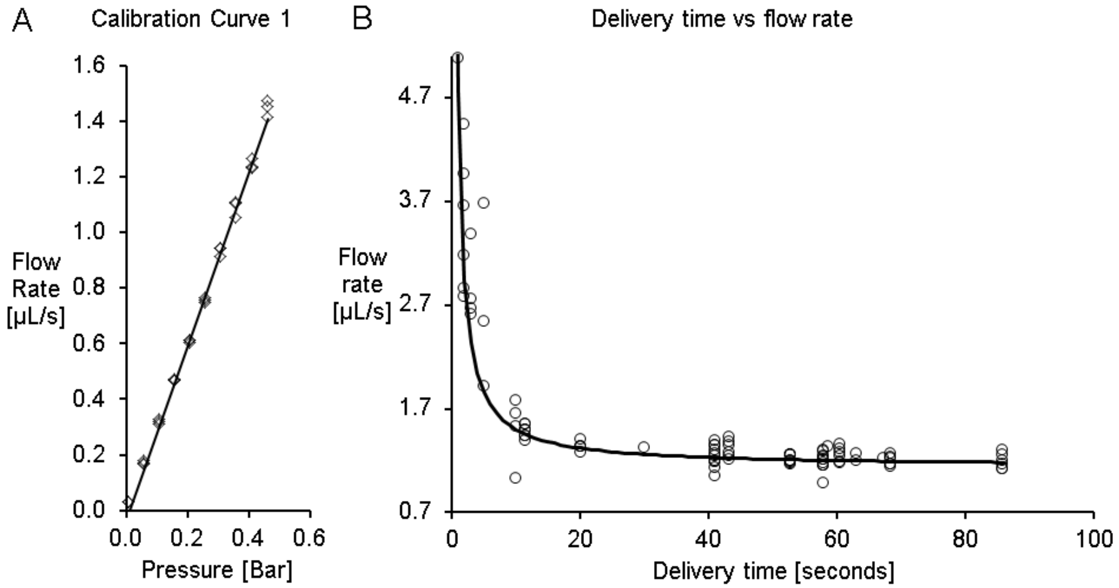


**Figure S5** – Calibration of the fluid delivery system. (A) The flow rate varies linearly with pressure for a fixed delivery time (least squares fit, $y=3.12x-0.03$, $R^2=0.99$). (B) The flow rate has a non-linear dependence on the delivery time, due to the elasticity of the C-Flex tubing. For an input pressure of 0.5 bar, the average flow rate is approximately $(3.61 + 1.14t)/t$, where $t$ refers to the delivery time, and flow rate is given in µL/s. These calibration curves enable precise delivery of fluid volumes to the microscope stage.
